# Personalised prostate MRI: tailoring contrast medium use in an era of increasing demand

**DOI:** 10.1007/s00330-025-11755-2

**Published:** 2025-06-16

**Authors:** Georgios Agrotis, Stephan Ursprung, Ivo G. Schoots

**Affiliations:** 1 Department of Radiology, Netherlands Cancer Institute, Amsterdam, The Netherlands; 2 American Medical Centre, Nicosia, Cyprus; 3 Department of Radiology, University Hospital Tübingen, Tübingen, Germany; 4 Department of Radiology and Nuclear Medicine, Erasmus University Medical Center, Rotterdam, The Netherlands

**Keywords:** MRI, Prostate, Gadolinium, Contrast media, Quality assurance

## Abstract

**Abstract:**

Prostate MRI has an established role in early diagnosis, staging, and active surveillance of prostate cancer (PCa), and its demand continues to rise. Moreover, the growing use of active surveillance for patients with low-risk and favourable intermediate-risk prostate cancer, along with ongoing research into MRI-based screening, is expected to further increase the need for MRI follow-up. Routine use of bi-parametric MRI and more selective, evidence-based administration of gadolinium contrast agents may help reduce the growing strain on MRI services. This special report explores scenarios in which contrast enhancement can improve diagnostic confidence, as well as situations where it may be safely omitted. It also examines the potential of on-table monitoring to guide patient-specific contrast administration, highlighting recent evidence suggesting this approach could reduce contrast use in three out of four men without compromising cancer detection or biopsy rates. Finally, the report outlines future research directions aimed at optimising scan times and contrast use in prostate cancer MRI.

**Key Points:**

***Question*** *With the growing demand for prostate MRI, how can we optimise scanner and specialist time, minimise gadolinium use, and still maintain high diagnostic accuracy*?

***Findings*** *Syer et al demonstrate the feasibility of using on-table monitoring to reduce the number of contrast-enhanced prostate MRI exams by 76%, without compromising cancer detection or biopsy rates*.

***Clinical relevance*** *While promising, the effectiveness of on-table monitoring for guiding contrast-enhanced acquisitions requires further validation. Tailoring imaging protocols to individual patient risk profiles will be essential for optimising resource use while maintaining diagnostic quality*.

Healthcare systems experience an increased demand for MRI services across various clinical scenarios, patients face longer waiting times and rising costs [1]. This worrisome trend may be reinforced when population-based screening programs for prostate cancer (PCa) are further investigated, as screening studies such as Göteborg-2 and STHLM3-MRI suggest that after initial PSA screening, a substantial proportion of men (7–12%) may require MRI [2, 3]. To address these challenges, the international PI-RADS steering committee has acknowledged the potential of non-contrast MRI as a strategy to alleviate system pressure [4]. They advocated for approaches such as patient recall and on-table monitoring in default non-contrast settings as safety nets, ensuring accurate diagnostic work-up with dynamic contrast-enhanced (DCE) MRI when indicated. Ultimately, maintaining the diagnostic quality of prostate MRI remains a priority, as healthcare systems strive to balance the demand with resource availability.

In their recent publication in *European Radiology*, Syer et al prospectively investigated the feasibility and efficacy of on-table monitoring for prostate MRI as a pathway to reduce the use of gadolinium-based contrast agents while increasing capacity and maintaining diagnostic accuracy [5]. This study is particularly relevant given the ongoing debate regarding the necessity of contrast use and DCE imaging in prostate MRI for cancer detection [6, 7].

The findings of this study support a more selective and personalised approach for contrast medium use in PCa diagnosis. The study suggests that contrast could be omitted in three-in-four men (76%) undergoing MRI examination without having a negative impact on biopsy rate or detection of clinically significant prostate cancer (csPCa). The substantial reduction in contrast use with on-table monitoring lowered costs, patient burden, and potential patient safety issues.

Syer et al found DCE imaging to be subjectively useful in two-in-three men (67%), however, they did not quantify how often this influenced clinical decisions [5]. On-table monitoring and subsequent DCE imaging improved reader confidence and diagnostic MRI quality, especially in men with metal works and artefacts from rectal gas and patient movements. Upgraded indeterminate MRI results (from PI-RADS 3 to 4 (3 + 1)) as a result of DCE imaging led to additional csPCa detection in some cases. Overall, the csPCa yield did not differ between the on-table and the matched non-on-table monitoring groups.

Further reasons to include DCE imaging include suspected locally advanced disease by clinical examination (cT3b/T4), accurate staging of seminal vesicle or bladder neck invasion for surgical candidates, and conditions other than adenocarcinoma of the prostate, such as mucinous cancers or abscess formation (Fig. 1). Evidently, DCE imaging should be excluded from men with contrast allergies or (end stage) renal failure.

**Fig. 1:**
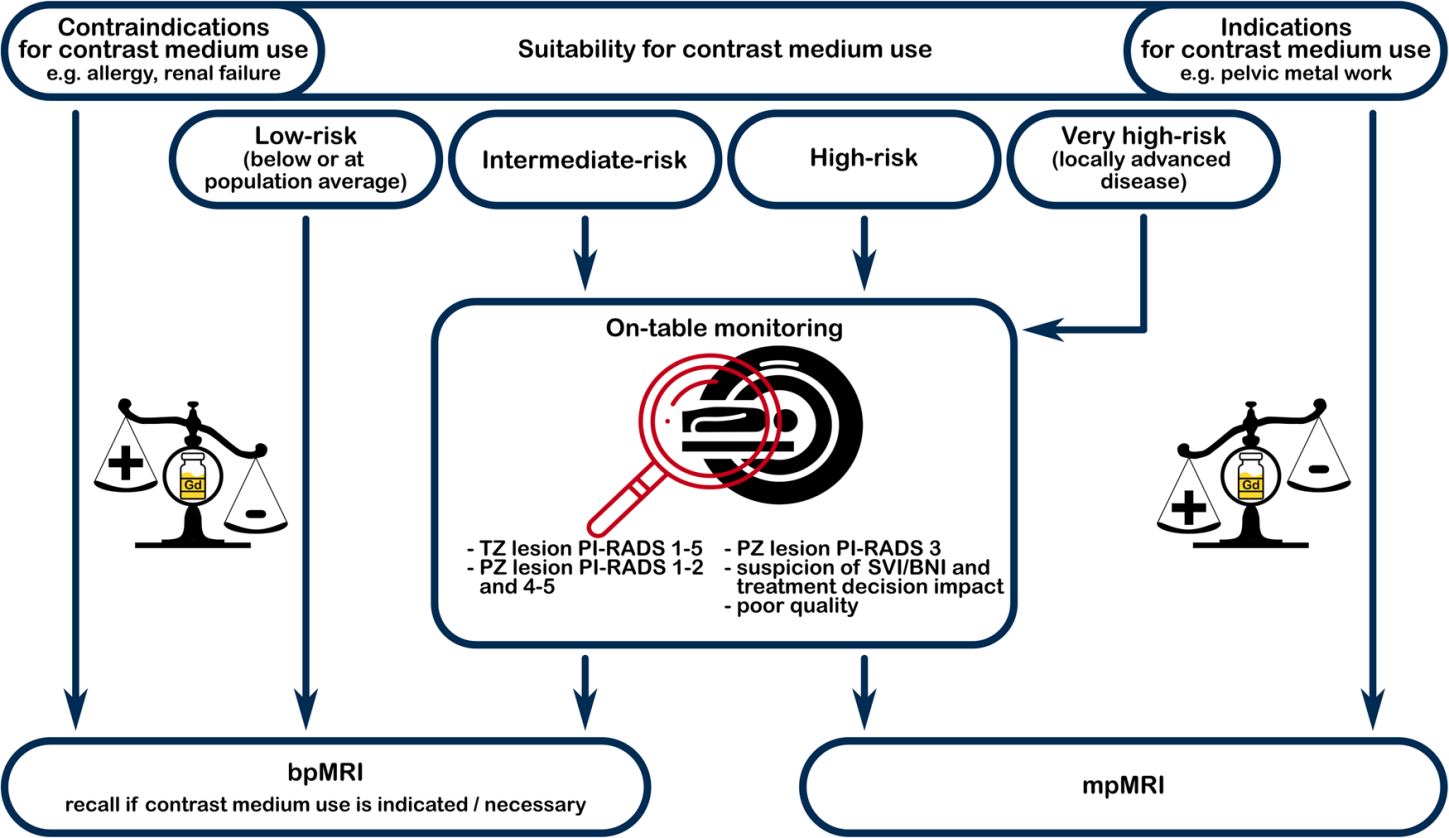
Personalised prostate MRI—tailoring contrast medium use. The on-table monitoring strategy of prostate MRI is a risk-adapted use of gadolinium-based contrast agents for PCa diagnosis, in addition to T2- and diffusion-weighted imaging. Low-risk men are unlikely to benefit from the use of contrast medium. Only a limited proportion of intermediate-, high-risk, and very high-risk men may benefit from the use of contrast medium, and on-table monitoring of prostate MRI may guide decisions on contrast medium use. BNI, bladder neck invasion; bpMRI, bi-parametric MRI; mpMRI, multiparametric MRI; PZ, peripheral zone; SVI, seminal vesicle invasion; TZ, transition zone

On-table monitoring for contrast use is one approach to resolve the challenging upfront decisions about whether to perform MRI with or without contrast. The advantages of personalised contrast utilisation in prostate MRI are multifaceted. Firstly, the direct cost savings associated with reduced contrast agent consumption and diminished intravenous cannula placements are considerable, particularly at scale. Secondly, minimising contrast exposure mitigates potential patient risks, albeit rare, associated with gadolinium administration. Furthermore, streamlining workflow by reducing the number of DCE-MRI acquisitions can enhance departmental efficiency and throughput, addressing rising demands, and potentially alleviating patient waiting times. Moreover, patient-tailored decisions, including personalised cancer-averse vs biopsy-averse strategies, will determine how MRI is best delivered. Beyond these direct benefits, a more nuanced consideration revolves around the environmental impact of gadolinium contrast agents. The increasing use of these agents contributes to environmental pollution, and a concerted effort to minimise their usage aligns with broader sustainability goals within healthcare.

Several challenges must be addressed before adopting the on-table monitoring strategy for contrast use. A primary concern is the availability of expert radiologists to make real-time decisions, particularly in settings that experience workforce shortages, increased exam volumes, and radiologist burnout. The necessity of an experienced radiologist potentially limits on-table monitoring during the daytime and outside core working hours. Patient throughput may be negatively impacted by scheduling challenges, on-table placement of intravenous cannulas, and provision of informed consent before the scan for contrast injection. Consequently, implementing a non-contrast MRI strategy with on-table monitoring requires multi-centre feasibility studies in different practice settings. Quality assurance of image acquisition and reporting within individual centres is essential. PI-RADS 3 lesions, which benefit the most from DCE imaging, are prone to significant inter-reader variability, an issue that requires further investigation. Future research should clarify patient features, disease characteristics, and image quality metrics associated with the clinical benefit of DCE-MRI. Future research should focus on refining patient-specific imaging strategies, rather than viewing biparametric MRI and multiparametric MRI as opposing approaches.

There are several advancements that could improve decision-making related to the use of contrast. AI-powered decision support systems for contrast applications could facilitate workflow efficiency gains, as highlighted by the impactful study of Syer et al [5]. These systems could also help reduce the burden on the already stretched radiology workforce, address the shortage of radiologists, increase throughput, and meet the growing demand for imaging by automating decisions related to contrast. Artificial intelligence, whether for interpretative or non-interpretative tasks, shows great potential in improving workflow efficiency [8].

The rising demand for prostate MRI will undoubtedly present logistical challenges within the radiological workflow. This underscores the need to work smarter by leveraging all available techniques to design efficient diagnostic protocols. Future research should prioritise adapting imaging strategies to patient risk profiles, including evidence-based contrast agent use, along with other strategies to improve efficiency and achieve the best possible diagnosis within the constraints of a healthcare system.
